# Efficacy of laparoscopic transabdominal preperitoneal (TAPP) and totally extraperitoneal (TEP) repair in the treatment of bilateral inguinal hernia: a systematic review and meta-analysis

**DOI:** 10.3389/fsurg.2026.1942077

**Published:** 2026-10-13

**Authors:** Yulin Pan, Xiao Pang, Pan Shen, Fenglan Liu

**Affiliations:** 1 Department of Surgical Care, Dazhou Central Hospital, Dazhou, Sichuan, China; 2 Department of General Surgery, Dazhou Central Hospital, Dazhou, Sichuan, China

**Keywords:** bilateral inguinal hernia, efficacy, meta-analysis, systematic review, TAPP, TEP

## Abstract

**Background and objective:**

Inguinal hernia is a common general-surgical disease. Laparoscopic repair is widely used for bilateral cases. The practical trade-offs between laparoscopic transabdominal preperitoneal (TAPP) and totally extraperitoneal (TEP) repair remain debated, and most existing publications focus on unilateral inguinal hernia.

**Methods:**

We searched multiple domestic and international databases for comparative studies of TAPP versus TEP for bilateral inguinal hernia. Literature retrieval covered records from database inception to April 6, 2026. Evaluated outcomes included general complications, special complications, recurrence, postoperative pain, operative time, and length of hospital stay. Statistical analyses were performed with RevMan 5.3 and Stata 18.

**Results:**

Nine eligible studies involving 1,319 patients were included (846 patients in the TAPP group and 473 in the TEP group). Pooled data show no significant between-group differences for general complications, special complications, postoperative pain, operative time, and length of hospital stay (all *P* > 0.05). The overall pooled result indicated a trend toward lower short-term recurrence with TAPP, which was largely driven by retrospective non-randomized cohort data. Although the RCT subgroup yielded a borderline nominally significant result, this signal derived from very limited trial data and does not establish a genuine between-group difference in recurrence risk. Given the maximum follow-up of 12 months across eligible studies, this recurrence-related finding warrants cautious interpretation.

**Conclusion:**

TAPP and TEP deliver comparable short-term safety and efficacy for bilateral inguinal hernia repair. The observed trend of lower short-term recurrence for TAPP relies heavily on observational cohort data and receives no support from RCT evidence. We cannot draw definitive conclusions about recurrence benefits. Further large-sample, long-term high-quality studies are needed.

## 1 Introduction

Inguinal hernia represents a common condition in general surgery. Bilateral disease accounts for 8%–30% of all inguinal hernia presentations (1, 2). Laparoscopic repair has become a preferred surgical option for patients with bilateral inguinal hernias. Compared with open surgery, laparoscopic approaches produce less postoperative pain and allow faster patient recovery (3). Published evidence supports satisfactory safety and effectiveness for simultaneous laparoscopic bilateral hernia repair (4). International hernia guidelines recommend laparoscopic or endoscopic techniques for bilateral inguinal hernia repair (5, 6). Two main laparoscopic techniques are widely used: laparoscopic transabdominal preperitoneal (TAPP) and totally extraperitoneal (TEP) repair. Both methods place mesh within the preperitoneal space, but they differ in surgical access. TAPP enters the peritoneal cavity before dissecting the preperitoneal plane. TEP completes the whole dissection within the extraperitoneal space without peritoneal puncture. Multiple primary studies have compared TAPP with TEP for bilateral inguinal hernia yet yielded inconsistent results. Heterogeneity may stem from variations in patient recruitment, surgical definitions, outcome assessment and sample size, with evidence limited to a mix of retrospective cohorts and few randomized controlled trials. Given insufficient direct head-to-head data, we conducted this meta-analysis to synthesise available evidence comparing the two techniques and inform future study design.

## 2 Methods

### 2.1 Inclusion and exclusion criteria

#### 2.1.1 Inclusion criteria

(1) All participants were diagnosed with bilateral inguinal hernia. (2) Studies compared TAPP versus TEP (TAPP as intervention and TEP as control, or vice versa). (3) Studies reported quantitative data for at least one prespecified primary or secondary outcome of interest in this meta-analysis.

#### 2.1.2 Exclusion criteria

(1) Studies with unavailable full text or identified as duplicates. (2) Non-comparative study designs, such as case reports, narrative reviews, conference abstracts, and dissertations. (3) Studies lacking a concurrent control group, reporting insufficient outcome data for quantitative synthesis, or assessed as high risk of bias according to Cochrane risk-of-bias tools.

### 2.2 Retrieval strategy

This meta-analysis follows the PRISMA 2020 Statement. We searched four electronic databases: PubMed, Cochrane Library, Embase and the Chinese Biomedical Database. Search coverage ran from database inception to April 6, 2026. We applied no language restrictions. Eligible study designs included randomized controlled trials and retrospective cohort studies. Search terms combined Medical Subject Headings and free-text keywords. Key search terms covered “Bilateral Inguinal Hernia”, “Inguinal Hernias”, “Transabdominal Preperitoneal”, “TAPP”, “Totally Extraperitoneal”, “TEP” and related variants. We also screened reference lists from retrieved papers to identify extra eligible records. All candidate papers went through full-text assessment against our predefined inclusion criteria.

### 2.3 Data extraction and quality evaluation

#### 2.3.1 Data extraction

Two independent reviewers completed literature screening and study selection following PRISMA guidelines. They resolved disagreements through group discussion. A third reviewer made the final judgement if consensus could not be reached. We first removed duplicate records. Next, we excluded non-comparative publications such as case reports, narrative reviews, conference abstracts and dissertations. Titles and abstracts were screened to rule out papers that clearly violated our inclusion or exclusion criteria. Remaining records underwent full-text eligibility assessment. We used a pre-tested standardized form to collect data. Extracted items included author information, publication year, country, study design, sample size, and baseline patient characteristics (age, sex, body weight, hernia type and laterality). Adverse events and recurrence were counted at the patient level. Recurrence was defined as any hernia relapse on either groin; one patient was counted as a single recurrent case even when only one side developed recurrence. We predefined recurrence as the single primary outcome for this review. Secondary outcomes included special complications, general complications, postoperative pain intensity, operative time, length of hospital stay and follow-up duration. Secondary-outcome definitions are described below. Special complications included scrotal swelling, hematoma, seroma, chronic groin pain ≥3 months, mesh infection, sensory disturbance, and intestinal obstruction occurring during hospitalization or throughout follow-up. General complications covered subcutaneous emphysema, pulmonary infection, venous thromboembolism, urinary retention, and surgical site infection occurring during hospitalization or within 7 days postoperatively. Postoperative pain intensity was measured via validated scales during hospitalization.

#### 2.3.2 Quality evaluation

Two reviewers independently assessed the methodological quality of included studies. For randomized controlled trials (RCTs), risk of bias was evaluated using the Cochrane Risk of Bias tool, assessing five domains: randomization process, deviations from intended interventions, missing outcome data, measurement of the outcome, and selection of the reported result. For non-randomized controlled studies (NRCSs), quality was assessed using the Newcastle-Ottawa Scale (NOS), with studies rated on selection, comparability, and outcome assessment. We defined an overall high-risk-of-bias judgement for a RCT when at least one domain was rated as high risk. Studies with high-risk domains were not automatically excluded from quantitative synthesis. Sharma et al. (9) was judged to carry a high risk of bias in the domain of blinding of participants and personnel. Yet its remaining four bias domains were assessed as low risk. The trial provided usable numerical data for our predefined endpoints. For this reason we retained it for meta-analysis, and this source of bias is noted during result interpretation.

### 2.4 Statistical methods

All meta-analytic computations were carried out in RevMan 5.3 (Cochrane Collaboration) and Stata 18. Weighted mean difference (*WMD*) or standardized mean difference (*SMD*) served as effect estimates for continuous outcomes; odds ratio (*OR*) was calculated for dichotomous data, with all effect sizes reported alongside their corresponding 95% confidence intervals. Between-study heterogeneity was evaluated using the *I^2^* statistic and Cochran's Q-test. We applied a fixed-effects model when heterogeneity was low (*I^2^* ≤ 50% and Q-test *P* ≥ 0.1). Where heterogeneity exceeded these thresholds, prespecified subgroup analyses were first undertaken to explore potential sources of inconsistency before adopting a random-effects model. Potential publication bias was examined via funnel-plot visual inspection, supplemented by Begg's rank-correlation test and Egger's linear-regression test. Leave-one-out sensitivity analysis was performed to test the robustness of pooled results. Statistical significance was set at *P* < 0.05.

## 3 Results

### 3.1 Literature search results

Our database search retrieved 561 records, with one additional publication identified by scanning reference lists. After duplicate removal, title-abstract screening and full-text eligibility assessment, nine studies ultimately met our inclusion criteria (Figure 1). This final set included four randomized controlled trials and five retrospective cohort studies, totalling 1,319 participants: 846 patients in the TAPP group and 473 in the TEP group. Baseline characteristics for all included studies are summarised in Table 1. Risk-of-bias outputs for RCTs are presented in Figures 2, 3; methodological quality for retrospective cohort studies was assessed with the Newcastle-Ottawa Scale, as shown in Table 2.

**Figure 1:**
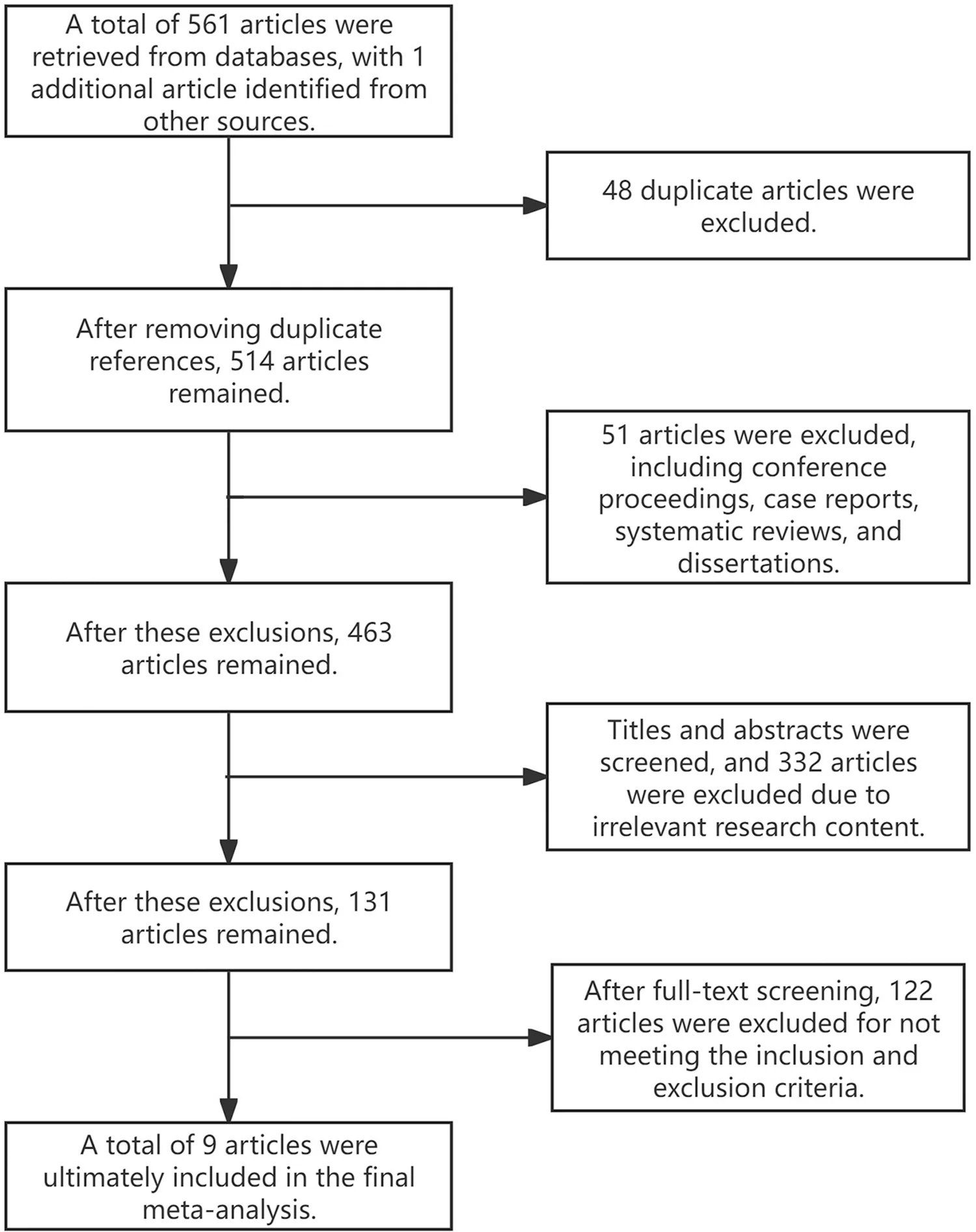
Study selection.

**Figure 2:**
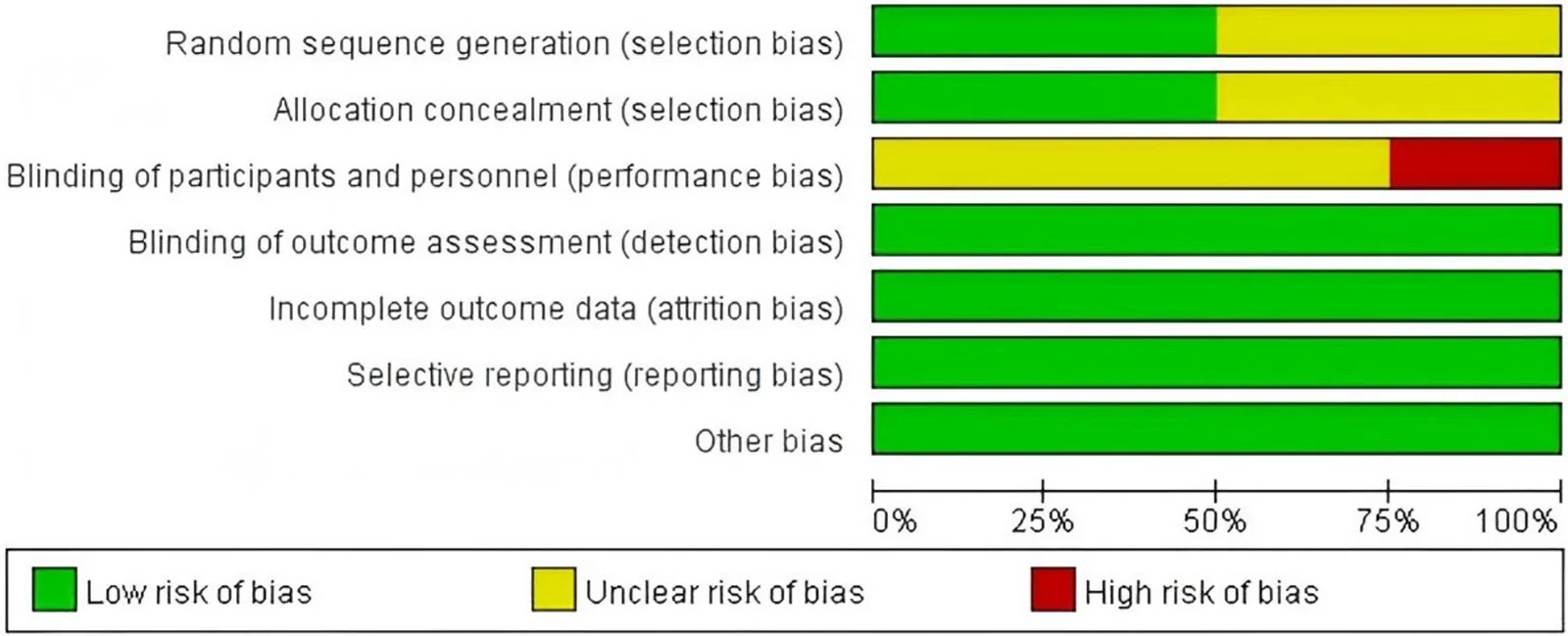
Risk of bias graph for the randomized controlled trials included in this study.

**Figure 3:**
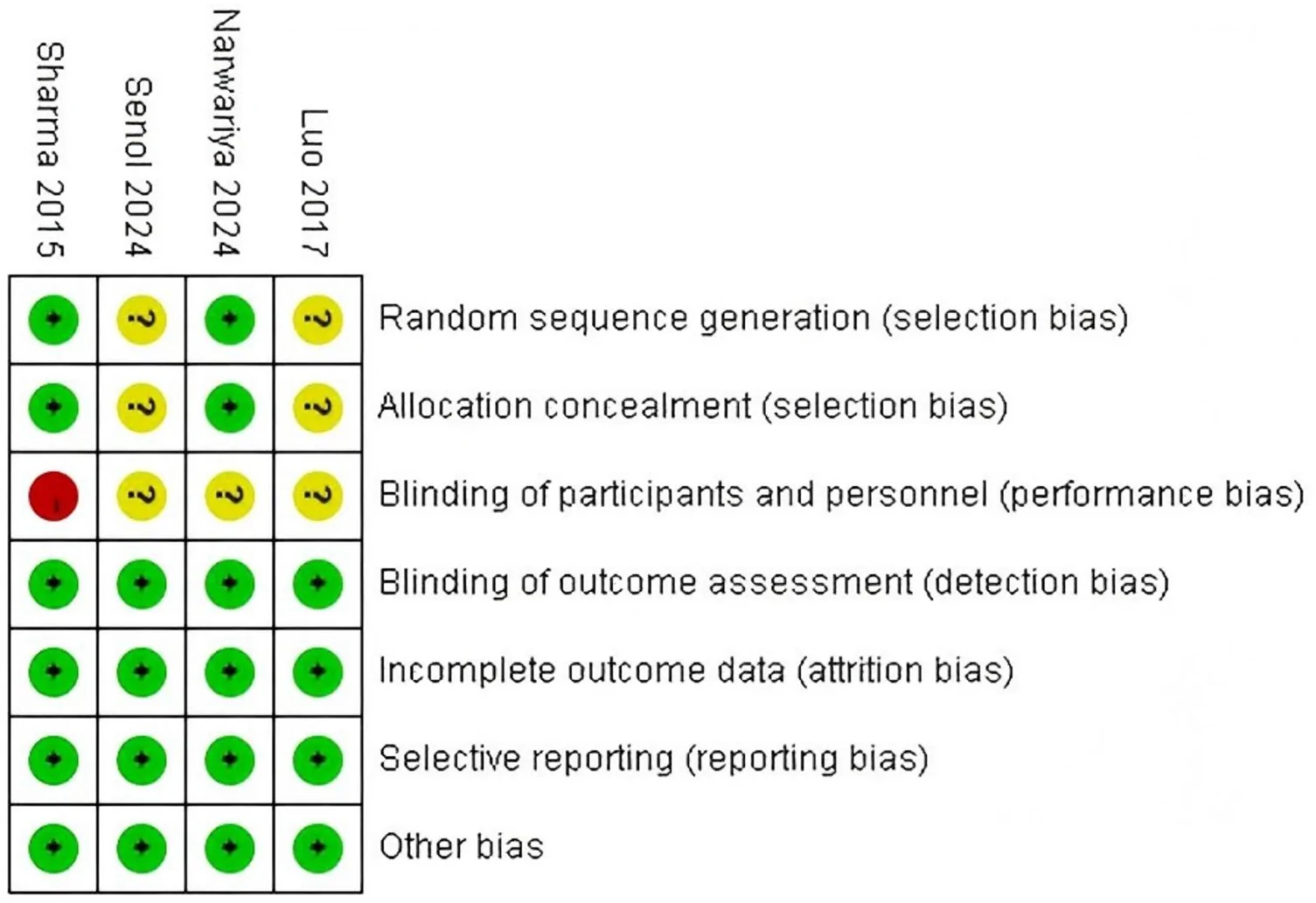
Risk of bias summary for the randomized controlled trials included in this study.

**Table 1:** Characteristics of the studies included.

Study	Country	Type of operation	Number of cases	Type of research	Age (years)	Gender [male (female)]	Outcome measures	Mesh fixation method
Zanghi et al. (7)	Italy	TAPP	305	NRCS	52	300 (5)	①②④⑤⑥	staple
		TEP	134		55	134 (0)		
Zeng et al. (8)	China	TAPP	54	NRCS	63.5 (22–93)	–	③⑤⑥	staple
		TEP	21					
Sharma et al. (9)	India	TAPP	30	RCT	49.4	59 (1)	①③⑤⑥	staple
		TEP	30		49			
Liu et al. (10)	China	TAPP	41	NRCS	58 ± 8.7	71 (5)	①②⑤⑥	Not fixed
		TEP	35		61 ± 7.6			
Luo (11)	China	TAPP	32	RCT	68 ± 10	26 (6)	①②④⑤⑥	–
		TEP	32		64 ± 14	28 (4)		
Hidalgo et al. (12)	Spain	TAPP	84	NRCS	62.44 ± 11.82	73 (11)	①②③④⑤⑥	adhesive
		TEP	71		64.54 ± 12.33	70 (1)		
Ozel and Kara (13)	Turkey	TAPP	200	NRCS	51.38 ± 8.22	188 (12)	①②④⑤⑥	Not fixed
		TEP	50		52.56 ± 10.83	47 (3)		
Senol (14)	Turkey	TAPP	50	RCT	57.2 ± 14.3	48 (2)	②④	–
		TEP	50		57.1 ± 14.5	47 (3)		
Narwariya and Kumar (15)	India	TAPP	50	RCT	49.87 ± 6.24	50 (0)	①②③④⑤⑥	Not fixed
		TEP	50		51.23 ± 6.09	50 (0)		

① General Complications ② Specific Complications ③ Pain ④ Recurrence ⑤ Operation Duration ⑥ Length of Hospital Stay.

**Table 2:** Quality evaluation of the retrospective cohort study by the Newcastle-Ottawa scale (NOS).

Study (year)	Selection	Comparability	Outcome	Total score
Zanghi et al. (2011) (7)	★★★	★★	★★	7★
Zeng et al. (2012) (8)	★★★	–	★★★	6★
Liu et al. (2016) (10)	★★★	★	★★	6★
Hidalgo et al. (2023) (12)	★★★	★★	★★★	8★
Ozel and Kara (2024) (13)	★★★	★★	★★	7★

### 3.2 Primary outcome measure

Six studies reported recurrence-related outcomes (7, 11–15). Luo (11) recorded zero recurrence events across both groups. Given zero-event cells for this rare endpoint, we applied the Peto odds ratio rather than the Mantel–Haenszel method for primary pooling. Heterogeneity was low (*I*^2^ = 38.5%, *P* > 0.1), so a fixed-effects model was implemented. The overall pooled Peto *OR* was 0.25 (95% CI = 0.11–0.57, *P* < 0.05) (Figure 4). This estimate pointed toward lower recurrence risk following TAPP repair. Subgroup stratification by study design exposed inconsistency across evidence types. Retrospective cohort data demonstrated a statistically significant reduction in recurrence for TAPP (Peto *OR* = 0.24, 95% CI = 0.09–0.69, *P* < 0.05). The RCT subgroup produced a Peto *OR* of 0.27 (95% CI = 0.08–0.99). This calculation drew from only two usable trials. Luo (11) was excluded from pooling owing to complete zero recurrence events in both arms. The upper bound of this confidence interval lies immediately below the null-effect value of 1. Randomized trials alone do not reach statistical significance for recurrence risk comparison between TAPP and TEP. The small sample base weakens confidence in this statistical signal. The overall pooled result is heavily weighted toward observational cohort data and should be treated as descriptive rather than conclusive. Sensitivity analysis using the Mantel–Haenszel approach produced consistent overall effect estimates (Figure 5). This finding confirms that our main result was not driven by statistical handling of zero-event cells.

**Figure 4:**
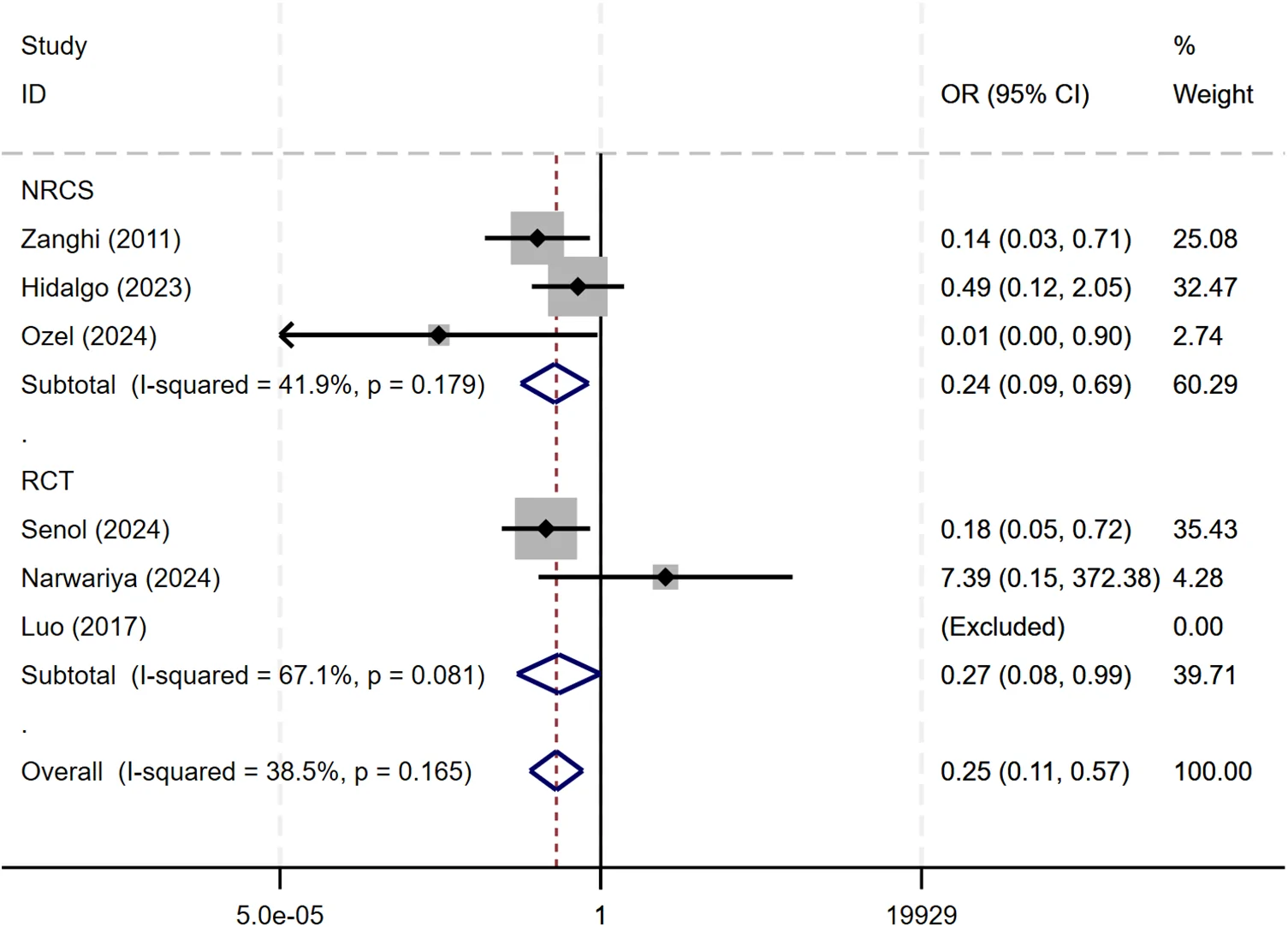
Comparison of the recurrence between the two groups (Peto).

**Figure 5:**
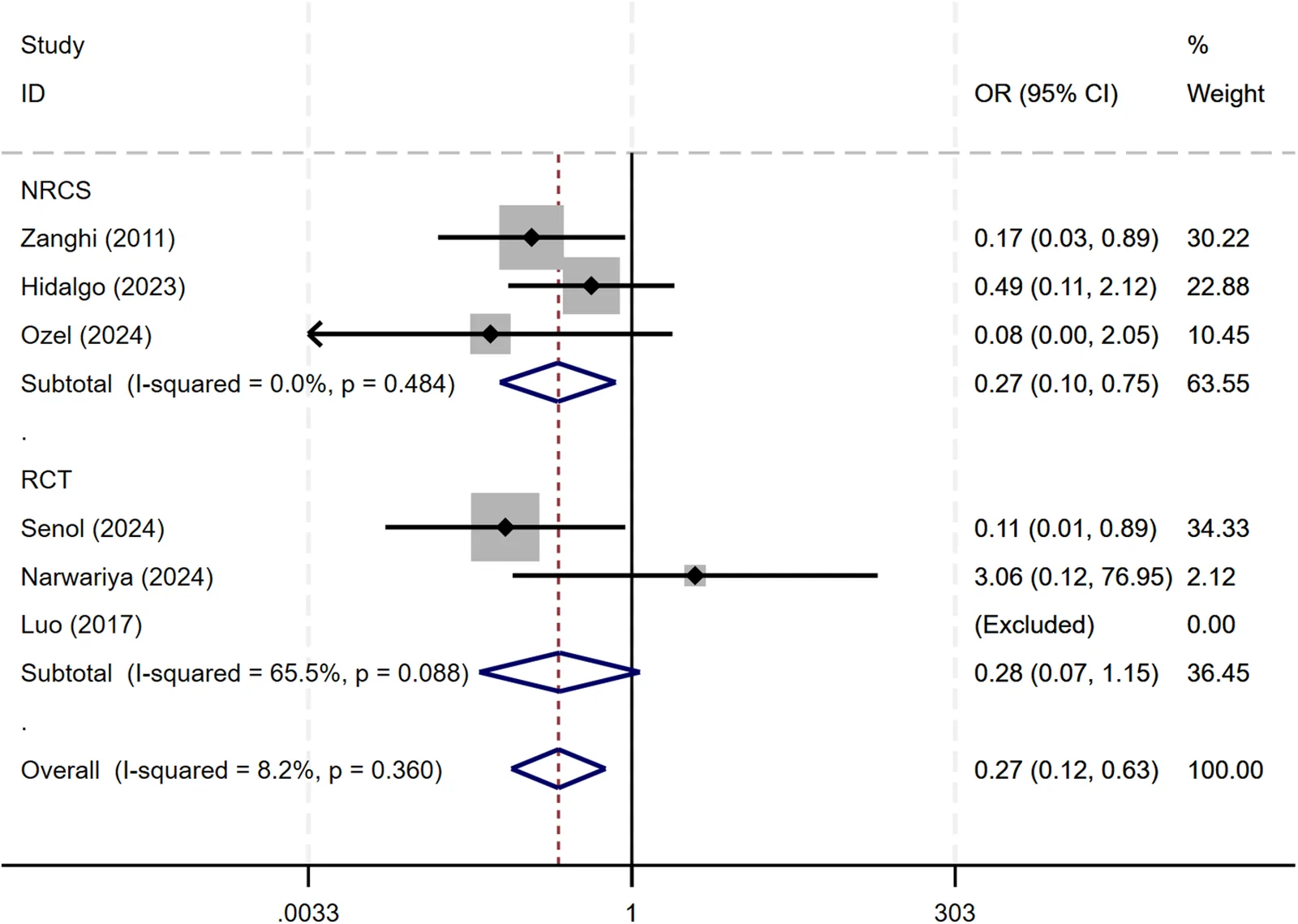
Comparison of the recurrence between the two groups (Mantel–Haenszel).

### 3.3 Secondary outcome measures

#### 3.3.1 Special complications

Specific complications unique to laparoscopic inguinal hernia repair were collectively defined as “special complications”. These included scrotal swelling, seroma, hematoma, chronic pain, mesh infection, mesh migration or shrinkage, and postoperative bowel obstruction. Seven studies reported data on these outcomes (7, 10–15). There was no statistical heterogeneity across studies (*I^2^* = 0%, *P* > 0.1), supporting the use of a fixed-effects model for pooled analysis. The combined estimate showed no significant difference in the overall risk of special complications between TAPP and TEP (*OR* = 0.88, 95% CI = 0.61–1.26, *P* > 0.05) (Figure 6). Subgroup analyses stratified by study design further demonstrated consistent null effects: in non-randomized comparative studies (NRCS) (*OR* = 0.98, 95% CI = 0.63–1.54, *P* > 0.05); in randomized controlled trials (RCTs) (*OR* = 0.70, 95% CI = 0.37–1.31, *P* > 0.05). No between-subgroup heterogeneity was detected, reinforcing the consistency of results across evidence tiers.

**Figure 6:**
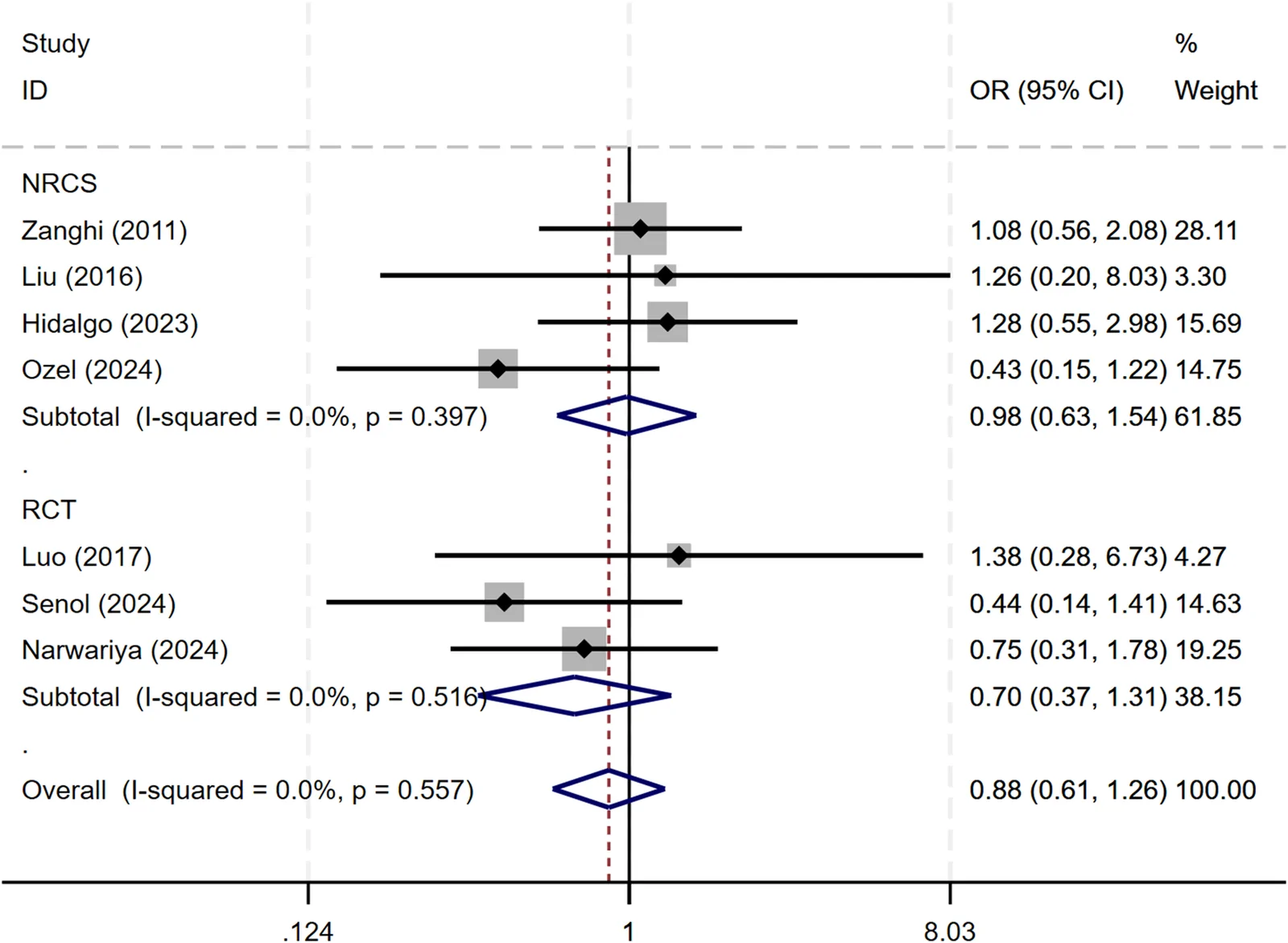
Comparison of the special complications between the two groups.

#### 3.3.2 General complications

General complications—defined as adverse events commonly observed across all laparoscopic procedures, including subcutaneous emphysema, pulmonary infection, venous thromboembolism, urinary retention, and surgical site infection—were pooled for analysis. Seven studies reported these outcomes (7, 9–13, 15). Significant heterogeneity was detected (*I^2^* = 64.8%, *P* < 0.1), warranting the use of a random-effects model. Meta-analysis revealed no statistically significant difference in the overall risk of general complications between the two surgical approaches (*OR* = 0.51, 95% CI = 0.19–1.36, *P* > 0.05) (Figure 7). Subgroup analyses stratified by study design further confirmed this finding: neither the non-randomized comparative studies (NRCS) subgroup (*OR* = 0.51, 95% CI = 0.12–2.11, *P* > 0.05) nor the randomized controlled trials (RCTs) subgroup (*OR* = 0.55, 95% CI = 0.16–1.89, *P* > 0.05) demonstrated a significant between-group difference.

**Figure 7:**
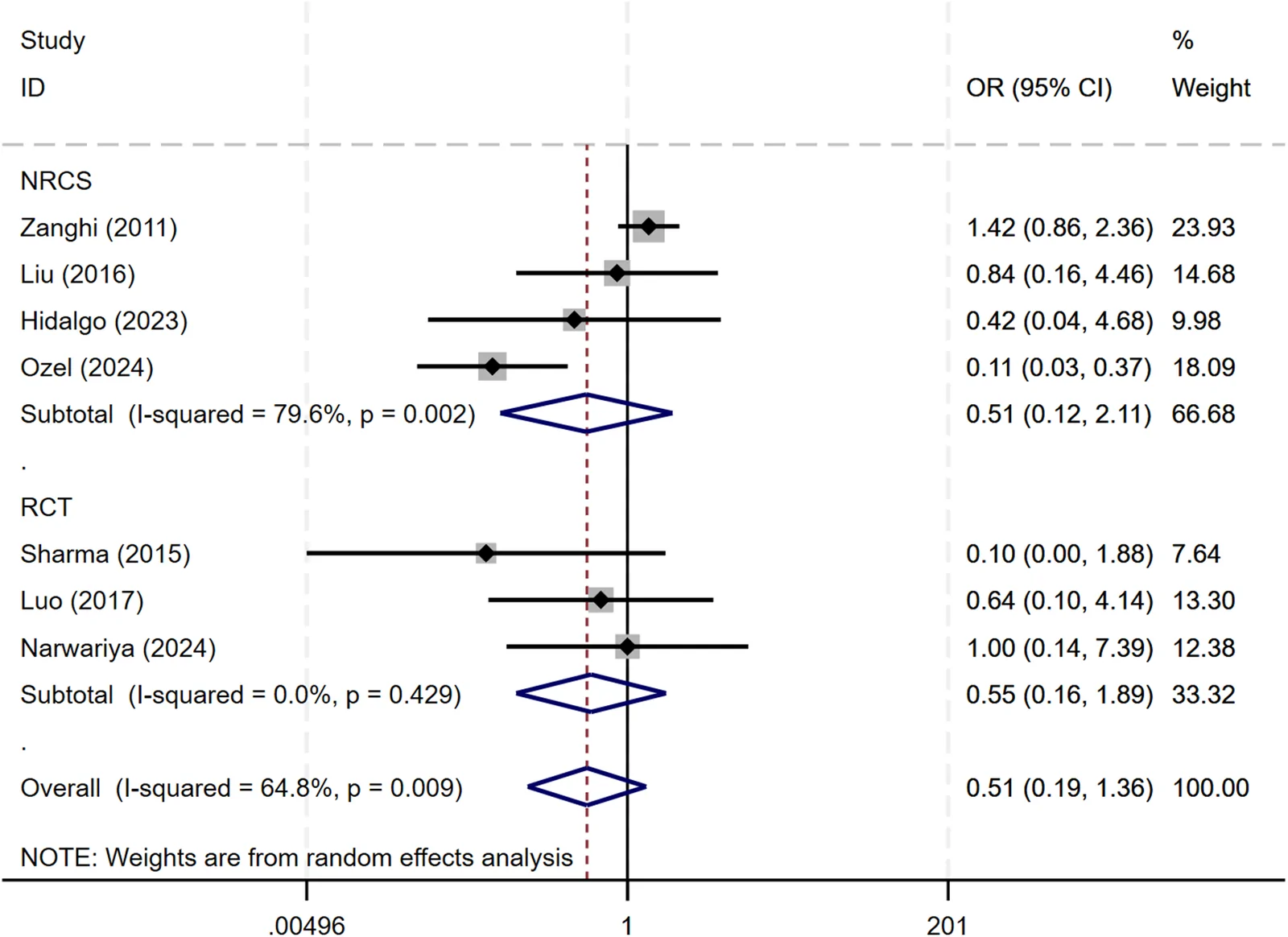
Comparison of the general complications between the two groups.

#### 3.3.3 Pain

Four studies reported postoperative pain using distinct assessment scales, with extreme overall heterogeneity detected (*I^2^* = 96.1%, *P* < 0.1). In light of such severe inter-study inconsistency, the unified overall pooled *SMD* is not emphasized in our analysis (Figure 8). Narwariya et al. (15) reported far less postoperative pain in the TAPP group. Zeng et al. (8) instead found a slight tendency toward milder pain with TEP. The other two trials found no meaningful difference between the two surgical techniques. These conflicting results stem from variations in pain scoring tools and differing intraoperative mesh fixation methods used in each study. To explore potential sources of heterogeneity, we excluded studies without mesh fixation and reran the analysis. After excluding the study that used no mesh fixation, heterogeneity among the remaining three studies decreased markedly (*I^2^* = 43.7%, *P* > 0.1), supporting the use of a fixed-effects model for this analysis. Pain scores remained comparable between groups (*SMD* = −0.01, 95% CI = −0.25–0.22, *P* > 0.05) (Figure 9).

**Figure 8:**
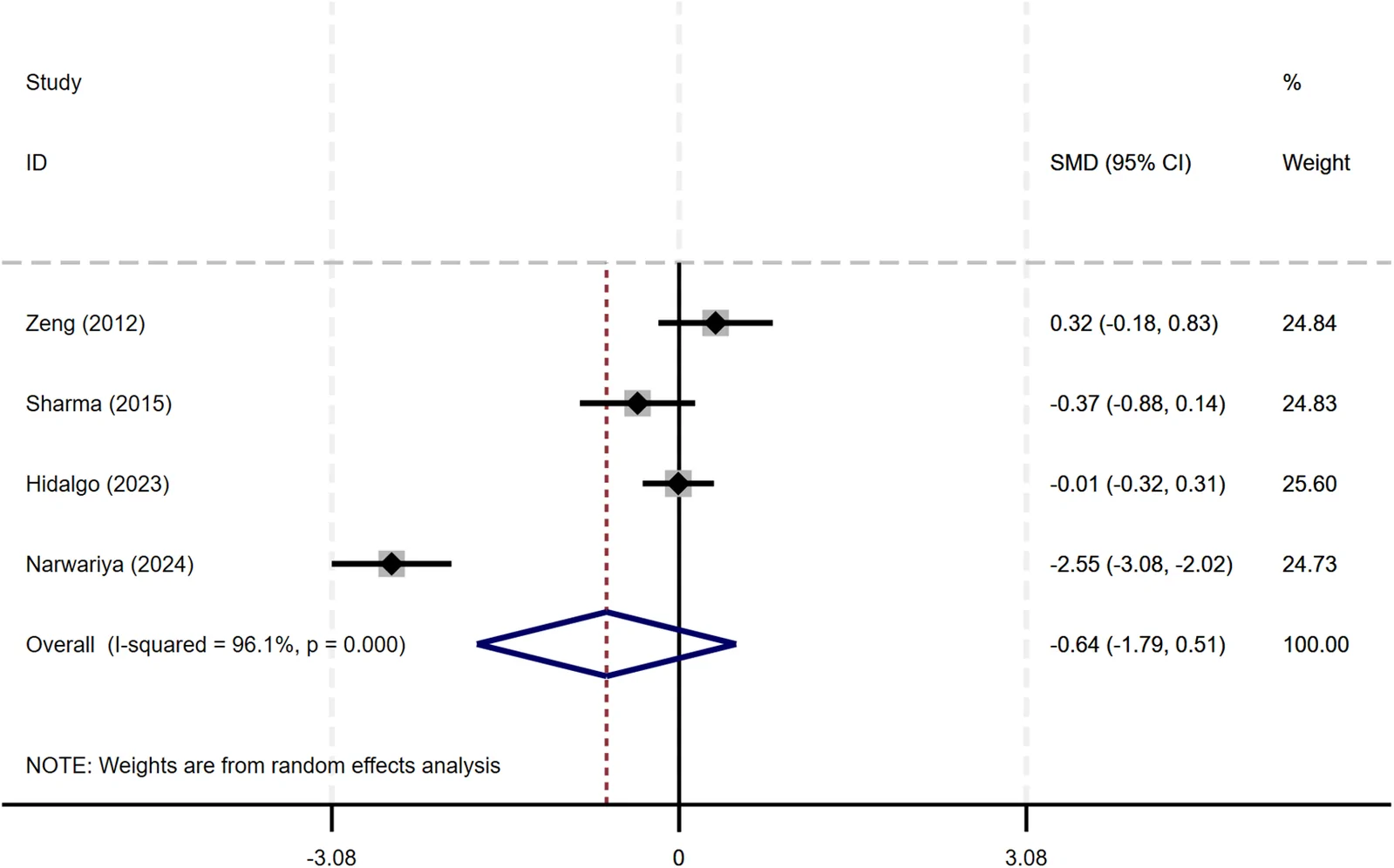
Comparison of the postoperative pain between the two groups.

**Figure 9:**
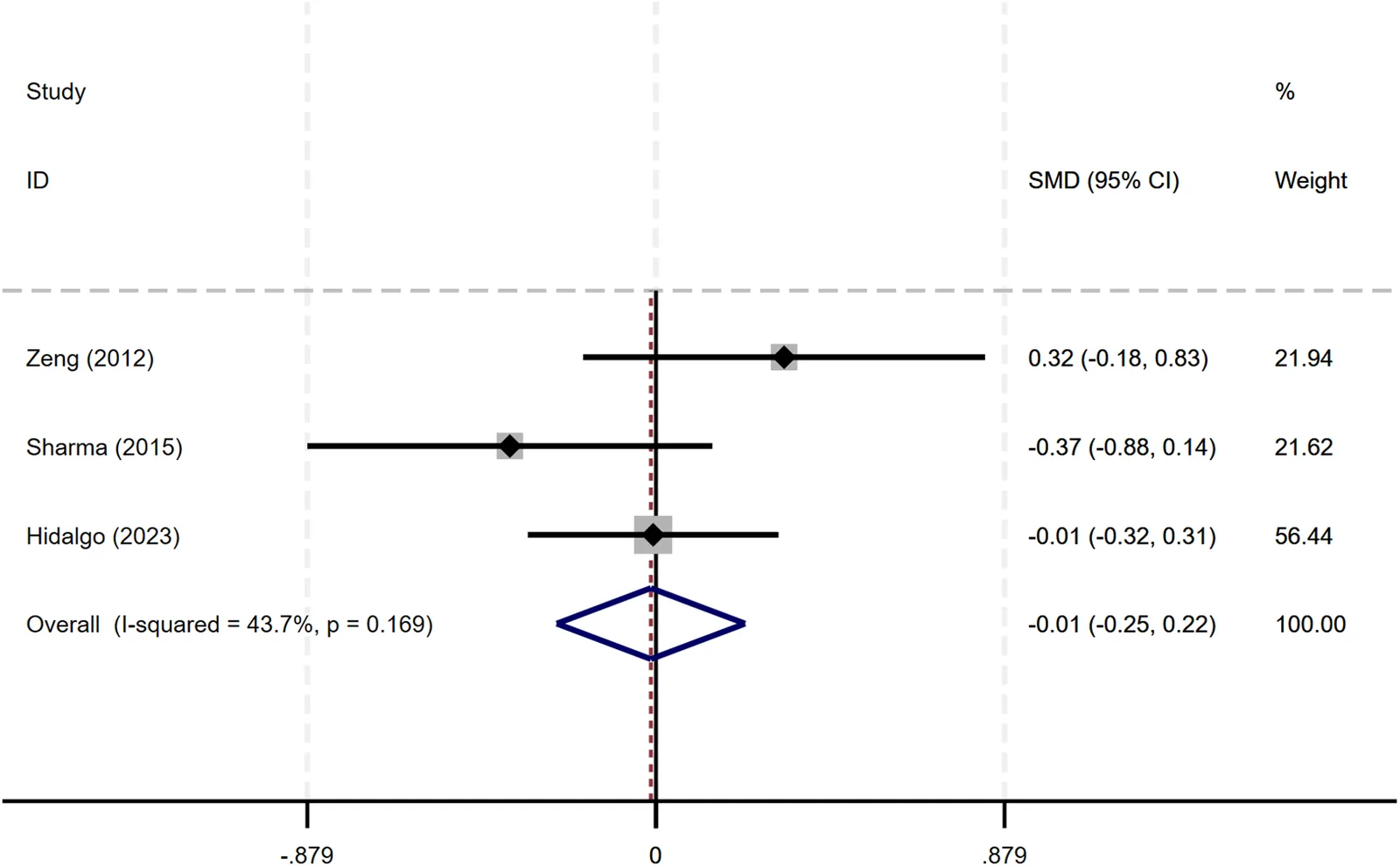
Comparison of the postoperative pain between the two groups with mesh fixation.

#### 3.3.4 Operation time

Eight studies reported operative time measured in minutes. We observed substantial inter-study heterogeneity across these records (*I^2^* = 99.3%, *P* < 0.1). Though we calculated a pooled *WMD* using the random-effects model (*WMD* = −9.48, 95% CI −31.64–12.67, *P* > 0.05) (Figure 10), this pooled estimate carries limited clinical value given the extreme inconsistency between individual studies, so we do not draw primary conclusions from this overall result. High heterogeneity persisted even when we split data by study design. For retrospective cohort studies, the pooled *WMD* was −11.98 (95% CI −38.71–14.75, *P* > 0.05); for RCTs, the pooled *WMD* was −5.21(95%CI −35.27–24.85, *P* > 0.05). Neither subgroup reached statistical significance. Individual trials showed conflicting operative-time findings. Zanghi et al. (7) and Ozel et al. (13) recorded shorter operating durations for TEP. Narwariya et al. (15), by contrast, reported longer operative times for TEP. The remaining four studies found little practical difference between the two approaches.

**Figure 10:**
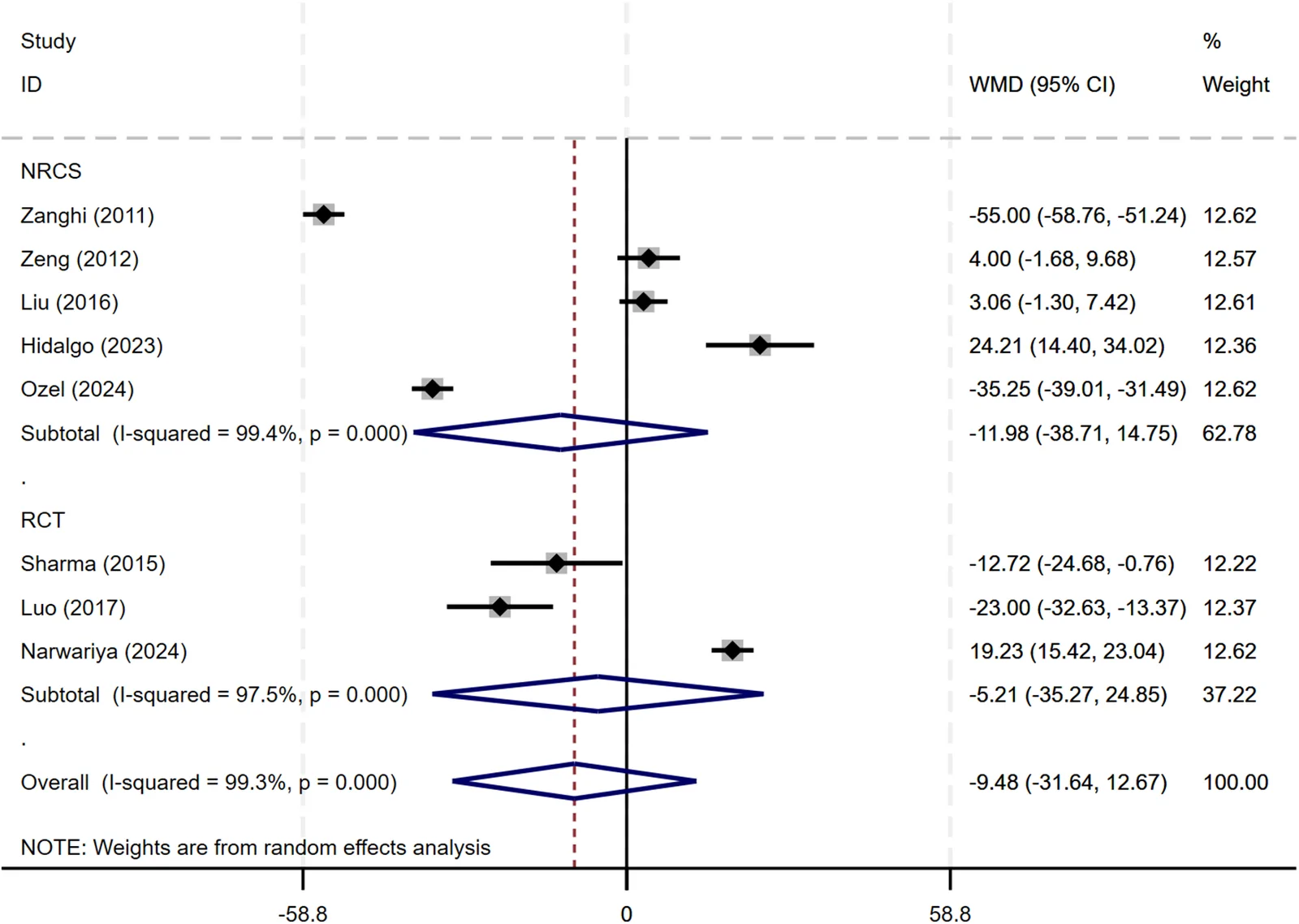
Comparison of the operation time between the two groups.

#### 3.3.5 Length of hospital stay

Seven studies reported postoperative hospital-stay duration measured in days. We detected substantial inter-study heterogeneity (*I^2^* = 87.1%, *P* < 0.1). The random-effects pooled *WMD* was 0.03 (95% CI −0.17–0.23, *P* > 0.05) (Figure 11). Given the severe inconsistency across included reports, this pooled estimate carries limited interpretive value and is not treated as core evidence in our analysis. Heterogeneity remained high even after stratifying by study design. For retrospective cohorts, pooled *WMD* was 0.12 (95% CI −0.28–0.53, *P* > 0.05). For RCTs, pooled *WMD* was −0.04 (95% CI −0.23–0.15, *P* > 0.05). Neither grouping yielded a statistically significant difference between TAPP and TEP.Individual trials produced conflicting hospital-stay outcomes. Hidalgo et al. (12) recorded longer hospitalisation following TEP repair, whereas Ozel et al. (13) observed shorter stays in the TEP group. Remaining studies found comparable lengths of stay for both surgical approaches.

**Figure 11:**
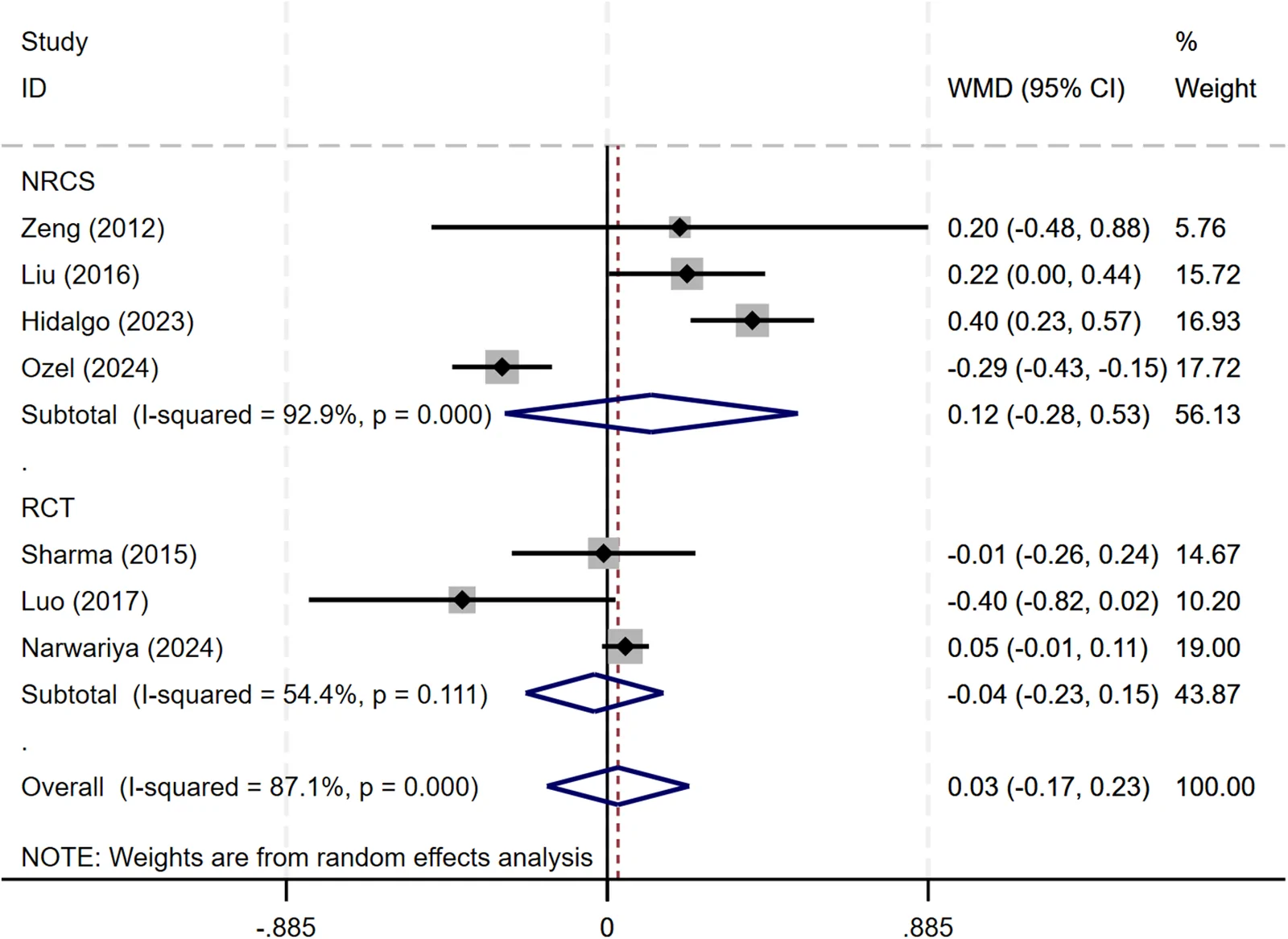
Comparison of the length of hospital stay between the two groups.

### 3.4 Publication bias and sensitivity analysis

We assessed small-study effects for recurrence, our predefined primary outcome. Visual inspection of the funnel plot showed scattered distribution of effect-size points across the plot (Figure 12). Formal testing yielded an Egger's test *P*-value of 0.648 and a Begg's test *P*-value of 1.000. Neither test indicated statistically significant evidence of publication bias. However, only six studies contributed data to this endpoint. Fewer than 10 included studies provides insufficient statistical power for reliable publication-bias evaluation. We therefore treat these results with caution and cannot draw firm conclusions about the presence or absence of small-study effects. Leave-one-out sensitivity analysis was performed to test the robustness of the pooled recurrence estimate (Figure 13). After sequentially removing each single trial, the overall effect size remained stable and no individual study reversed the direction of the pooled association. This pattern suggested that no single study exerted an overwhelming influence on the final pooled result.

**Figure 12:**
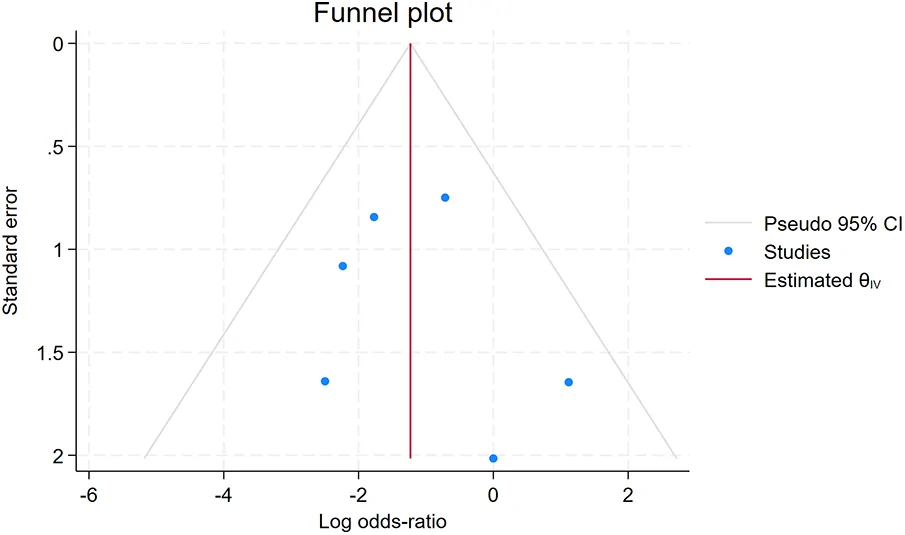
Funnel plot of the recurrence.

**Figure 13:**
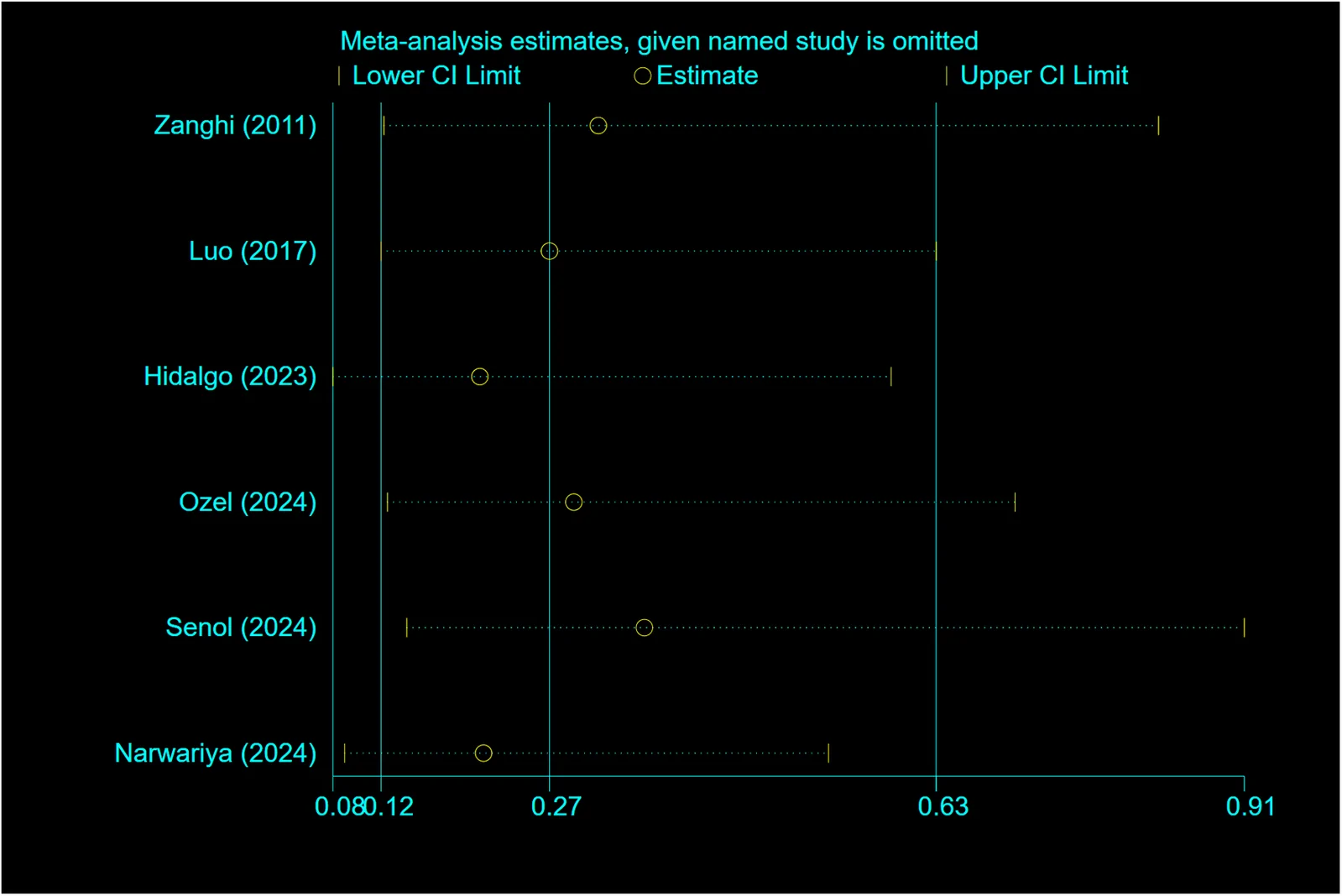
Sensitivity analysis of the recurrence.

## 4 Discussion

Laparoscopic inguinal hernia repair is widely recognized as the preferred method for bilateral inguinal hernias (14). It brings multiple clinical benefits, including milder postoperative pain, faster functional recovery, lower surgical site infection rates, and shorter hospital stay (3, 16, 17). The European Association for Endoscopic Surgery (EAES) accordingly recommends two mainstream laparoscopic techniques, transabdominal preperitoneal (TAPP) and totally extraperitoneal (TEP) repair, as first-line surgical options for bilateral inguinal hernia treatment (18). Existing meta-analyses and large cohort data support that shifting from open to laparoscopic repair does not raise rates of intraoperative harm, postoperative complications, or long-term recurrence (19).

When interpreting recurrence outcomes, readers should pay close attention to conflicting results between observational-cohort and RCT-derived estimates. Pooled data from retrospective cohorts suggested lower recurrence rates following TAPP repair. This association cannot be regarded as definitive clinical evidence. Observational studies carry inherent selection imbalance, and they contribute heavily to the overall pooled effect. The corresponding RCT subgroup returned a borderline 95% confidence interval of 0.08–0.99. Its upper limit sits just below the null-effect value of 1. Although this value meets conventional statistical significance, it comes from only two usable randomised trials after excluding one zero-event study. Marginal significance obtained from small-sample analyses is vulnerable to random chance. It cannot confirm true differences in real-world recurrence risk between TAPP and TEP. This inconsistency across study designs may partly stem from TEP's steep learning curve (20). Surgeons with limited experience may struggle to fully expose critical preperitoneal landmarks. These structures include the iliopubic tract, Cooper's ligament, and inferior epigastric vessels. Poor exposure can lead to suboptimal mesh positioning, incomplete defect coverage, intraoperative mesh folding or displacement, and compromised mechanical stability of the repair (21). Standardised TEP technique requires flat, tension-free mesh placement. The mesh needs to fully cover the myopectineal orifice to ensure even mechanical support. It should sit 0.5 cm away from the peritoneal margin. Dissected or ligated hernia sacs are positioned in front of the mesh. Standard large-pore mesh helps distribute intra-abdominal pressure effectively. It may lower hernia recurrence risk and also reduce the chance of seroma formation to some extent (22).

No measurable difference emerged between TAPP and TEP in the incidence of specific complications including scrotal swelling, haematoma, seroma, and mesh-related infection. Subgroup analyses yielded consistent results across study designs. This similarity may partly reflect growing standardisation of preperitoneal-space dissection and perioperative complication-prevention strategies in laparoscopic hernia surgery. Surgeons rely on sound knowledge of key anatomical landmarks such as the myopectineal orifice, corona mortis and the “danger triangle” to reduce operative injury. Technical refinements, such as transecting large hernia sacs instead of forceful extensive dissection, help limit vascular trauma and lymphatic disruption. For bilateral direct hernias, inversion and suture-closure of pseudosacs are commonly performed. For indirect hernias with an internal ring larger than 2 cm, primary ring closure is recommended. These manoeuvres can lower seroma risk. Strict adherence to sterile technique remains critical for avoiding mesh infection. Broad-use of large-pore meshes and limited application of fibrin glue are recommended, with single-strand sutures preferred where fixation is necessary. Such practices align with best-practice evidence derived largely from unilateral-hernia studies. Even so, sample size and relatively short follow-up within our included literature cannot support firm conclusions about long-term complication risks. Large-scale multicentre RCTs with extended follow-up (>5 years) are still needed to verify these findings.

We found no inter-group difference in general complications including subcutaneous emphysema, urinary retention and surgical-site infection. In RCT subsets, heterogeneity remained low (*I^2^*< 25%), supporting the stability of this observation. By contrast, non-randomised cohorts showed considerable heterogeneity, which may stem from inconsistent complication definitions, reporting standards or baseline patient characteristics across those studies. Ozel et al. (13) reported lower complication rates for TAPP, yet this single-study outcome was not mirrored in our pooled estimates. This discrepancy underscores the risk of drawing clinical conclusions from isolated study results. Limited RCT quantity and residual heterogeneity mean high-quality multicentre trials with unified complication definitions and follow-up protocols are still required to better compare real-world safety profiles. Subcutaneous emphysema is usually self-limiting and resolves within 24 h after surgery. Careful trocar placement helps reduce its occurrence, particularly for TEP, where misplaced optical ports may create unwanted subcutaneous dissection planes. Urinary retention represents one of the common early postoperative events after laparoscopic inguinal hernia repair, and bilateral hernia cases appear more vulnerable. Prolonged operative time, peritoneal insufflation-related bladder irritation and elevated sympathetic tone may contribute to this complication. Advanced age, male sex and pre-existing benign prostatic hyperplasia are recognised risk factors (23).

Some earlier studies suggested lower postoperative pain after TEP repair (24). Our pooled analysis, however, detected no meaningful difference in validated pain scores between TAPP and TEP. This finding aligns with a recent high-quality meta-analysis reported by Aiolfi et al. (25). Comparable pain outcomes may partly reflect the improved safety profile of modern-day TAPP and TEP techniques (26). Acute postoperative pain appears to be driven more by local inflammatory response than direct mechanical nerve or soft-tissue injury (22). Technical updates have narrowed historical gaps in pain risk. Suture-free mesh fixation, for instance, is now widely adopted for TAPP when hernia defects measure ≤3 cm. This practice helps reduce iatrogenic nerve injury and subsequent chronic groin pain (27). For larger defects requiring fixation, surgeons limit anchoring points to safe anatomical zones to avoid neurovascular damage (28, 29). Current clinical consensus discourages routine mesh fixation for indirect hernias during TEP, regardless of defect size (30, 31). Widespread use of lightweight large-pore meshes and standardized intraoperative nerve-preserving manoeuvres may further lessen differences in nerve-related adverse events between the two approaches. Past concerns about higher pain after TAPP mainly centred on staple-related nerve trauma (32). Our sensitivity analysis, performed after adjusting for mesh-fixation approaches, yielded similar pain profiles for both operations. This observation implies that operative technique, rather than the choice between TAPP or TEP itself, largely determines postoperative nociceptive burden.

We found no statistically significant difference in pooled operative time between TAPP and TEP, yet substantial residual heterogeneity remained even after stratifying studies by research design. Individual papers reported conflicting operative-time findings: Hidalgo et al. (12) and Nethaji et al. (33) described time-saving advantages for TEP attributed to single-port contralateral dissection and avoidance of peritoneal suture closure (12, 34), whereas Ozel et al. (13) noted longer setup time for preperitoneal space creation and frequent peritoneal tear repair that offset potential TEP benefits. Such discrepancies likely stem from uneven surgeon expertise, institutional procedural conventions, and variable management of complex bilateral hernias. Although we report pooled weighted mean differences for this endpoint, these summary statistics carry limited clinical reliability and cannot form the basis for firm clinical judgements, meaning future multicentre prospective trials adopting uniform skin-to-skin timing endpoints and predefined intraoperative complication protocols are needed to clarify real-world differences in operative duration.

Our meta-analysis detected no statistically significant difference in pooled postoperative length of hospital stay between TAPP and TEP for bilateral inguinal hernia repair. Opposing effect estimates emerged among non-randomised cohorts. Hidalgo et al. (12) recorded prolonged hospitalisation after TEP, while Ozel et al. (13) reported shorter stays for patients receiving TEP. These divergences most likely reflect variable institutional discharge criteria, local ERAS implementation, preoperative risk stratification and baseline comorbidity burden, rather than inherent differences between the two surgical techniques. The RCT subgroup yielded effect sizes clustered close to the null value and showed markedly lower heterogeneity. This finding supports comparable recovery trajectories under controlled trial conditions. High overall heterogeneity, however, limits the precision and generalisability of our pooled estimate. Future multicentre randomised trials should standardise not only surgical technique but also peri-operative care pathways.

Beyond our comparison of standard TAPP and TEP, enhanced-view totally extraperitoneal repair (eTEP) is a technical variant of conventional TEP. Surgeons adjust port positions to create a larger working surgical field. This modified technique has seen increasing use for preperitoneal hernia operations, especially in patients with large or complex groin hernias (34). Observational studies have identified several potential advantages of eTEP over standard TEP. These may include shorter operative time, less postoperative pain and a shorter hospital stay. Some reports also record higher seroma-related complication rates after conventional TEP (33, 35). Other real-world data, however, show no meaningful differences in short-term outcomes among TAPP, conventional TEP and eTEP. A small number of single-centre studies have even found eTEP to produce better results than TAPP (36). No studies evaluating eTEP satisfied our pre-set inclusion criteria. For this reason, our meta-analysis cannot provide a quantitative comparison of these three laparoscopic techniques. eTEP offers a valuable option for operations limited by the narrow working space of classic TEP.

## 5 Conclusions

Our meta-analysis shows TAPP and conventional TEP deliver comparable short-term safety and efficacy for bilateral inguinal hernia repair. Surgeons should individualise operative selection according to their own technical familiarity, local hospital resources, and patient-specific features, including anatomical conditions, comorbidity burden, and personal preference. TAPP may serve as a more accessible starting option in training-oriented or low-resource settings given its gentler learning curve and surgeons’ existing familiarity with transabdominal laparoscopic approaches. Operators can gradually transition to TEP once adequate procedural confidence and teamwork have been established. This work carries several notable limitations. Less than half of included studies were randomised controlled trials which leaves residual risk of selection bias and restricts causal inference. Follow-up duration across available literature never exceeded 12 months so we cannot draw conclusions for late endpoints such as chronic groin pain mesh-associated adverse events and long-term recurrence. Prespecified subgroup analyses failed to fully resolve high statistical heterogeneity for certain outcomes. Unmeasured variables such as annual surgeon caseload and mesh product characteristics may account for remaining inconsistency. The larger sample size within the TAPP arm reflects real-world clinical practice patterns rather than study inclusion bias. This marked sample-size imbalance shifts pooling weight toward TAPP data and may overestimate its apparent benefit for short-term recurrence.

## Data Availability

The datasets presented in this study can be found in online repositories. The names of the repository/repositories and accession number(s) can be found in the article/Supplementary Material.
